# Eating behavior of adolescent girls in countries with a high prevalence of stunting under five: a systematic review

**DOI:** 10.3389/fpsyg.2023.1228413

**Published:** 2023-11-27

**Authors:** Arlette Suzy Setiawan, Arief Budiarto, Ratna Indriyanti

**Affiliations:** ^1^Department of Pediatric Dentistry, Faculty of Dentistry, Universitas Padjadjaran, Bandung, West Java, Indonesia; ^2^Faculty of Psychology, Universitas Jenderal Achmad Yani, Bandung, West Java, Indonesia

**Keywords:** adolescent girls, eating behavior, nutrition, stunting, body image

## Abstract

**Background:**

Adolescence is critical for physical and psychological growth, making healthy dietary behavior essential. Several countries face nutritional challenges due to a high prevalence of stunting in children under five, which can impact adolescent girls’ eating behavior. This systematic review aims to provide evidence on the eating behavior of adolescent girls in such countries, offering insights for stunting prevention programs.

**Methods:**

We conducted a systematic review following PRISMA guidelines and registered it in PROSPERO. We employed a comprehensive search strategy across multiple databases. Study selection involved three stages: deduplication, title/abstract screening, and full-text review, with inter-reviewer agreement assessment.

**Results:**

The search identified 15 eligible studies from various countries, primarily utilizing cross-sectional designs. The studies focused on age ranges within adolescence and varied in sample size and assessment tools. The primary objective of these studies was to assess eating behavior, with outcomes related to dietary patterns, eating disorders, body image, and nutritional knowledge. The results revealed diverse eating behaviors among adolescent girls, including restrained eating, dieting for weight loss, craving-induced eating, and unhealthy dietary patterns. Many girls exhibited low dietary diversity, contributing to micronutrient deficiencies.

**Suggestions:**

To address these issues effectively, stunting prevention programs and policies should prioritize the following strategies: implementing comprehensive nutritional education programs to enhance adolescents’ knowledge of healthy eating habits and dietary diversity, providing resources and support for positive body image development to reduce the pressure for unhealthy dieting, improving access to affordable, nutrient-rich foods in both urban and rural areas, raising awareness about eating disorders and emphasizing early identification and intervention, engaging parents, caregivers, and communities in promoting healthy eating behaviors, and maintaining rigorous research and monitoring to adapt strategies based on evolving trends in adolescent eating behaviors.

**Conclusion:**

Adolescent girls in countries with high stunting prevalence exhibit diverse eating behaviors that can impact their nutritional status and overall health. Addressing these behaviors is essential to prevent stunting and its long-term consequences, emphasizing the importance of comprehensive strategies and support for this vulnerable population.

**Systematic review registration:**

https://www.crd.york.ac.uk/prospero/#recordDetails, identifier CRD42023389909.

## Introduction

The high stunting rate in children under five persists worldwide. Globally, 149.2 million children under five suffered from stunting in 2020 (World Health Organization, 2021). This number can increase given the limited nutritional intake during the COVID-19 pandemic, the impact of which is stunting, which may only manifest itself in the next few years (Aditri et al., 2022). The World Health Organization has set a maximum tolerance limit for stunting for toddlers at 20% (World Health Organization, 2021). Talking about malnutrition is often associated with children who do not get enough food. However, the reality in Indonesia and several other countries is more complex. Teenagers face a nutritional crisis (Mahriani et al., 2022). Adolescence is a nutritionally vulnerable period when rapid physical growth increases nutritional needs. Dietary behaviors formed in adolescence can contribute to nutrition-related problems that have long-term health consequences (Lassi et al., 2017; Mahriani et al., 2022).

The UN defines adolescents as those aged between 10 and 19, comprising 1.2 billion people worldwide or approximately 16% of the global population. Most of these adolescents live in low- or middle-income countries and face various social and economic challenges. Investing in the rights and development of adolescents can contribute significantly to their full participation in society, a competitive workforce, sustainable economic growth, better governance, and more dynamic civil society. It can also accelerate progress toward achieving the Sustainable Development Goals (SDGs) set by the United Nations (UNICEF, 2018).

Adolescents are increasingly seen as a window of opportunity with the recognition that investment in the health and wellbeing of young people is crucial for a country’s future and overall development. Evidence shows that adolescence provides a second chance to influence developmental trajectories (including cognitive growth and development), shape future habits, and compensate for some negative childhood experiences - second only to early childhood (UNICEF, 2018; United Nations Children’s Fund, 2021). Investing in adolescents protects future generations of adults and consolidates investments in early childhood health, survival, and education.

Malnutrition, in all its forms, can have long-term detrimental effects on the development of children - and can even be passed down from generation to generation. In addition to poor cognitive performance and low work productivity, adolescent girls with anemia, for example, are more likely to give birth to low birth weight babies at risk of growth stunting (Setiawan et al., 2022). In countries with a high prevalence of stunting, adolescent girls may face additional challenges in their eating behavior. Several factors that may contribute to this include: first, limited access to nutritious food - adolescent girls may live in areas where access to nutritious food is limited, resulting in a lack of essential nutrients and an increased risk of malnutrition. Second, poverty–adolescent girls who live in poverty may not have the resources to buy varied and nutritious food, leading to food insecurity and malnutrition. Third, beliefs and cultural practices–adolescent girls may be subject to beliefs and cultural practices that limit their food choices, such as food taboos and gender-based food consumption patterns. Fourth, lack of education–adolescent girls may not have access to education on nutrition and healthy eating habits, resulting in poor food choices. Lastly, time constraints–adolescent girls in countries with high stunting prevalence may have to spend much time on household chores, leaving them with little time to prepare food, which can lead to skipping meals or consuming fast food.

Healthy habits developed during adolescence, such as healthy eating and physical activity, can last a lifetime and help break the cycle of intergenerational malnutrition (Frech, 2012). However, programs aimed at improving nutrition during these formative years are still too few, especially concerning preventing stunting, so they must be quickly increased. Therefore, a study was conducted to evaluate the eating behavior of adolescent girls in several countries with a high prevalence of stunting in young children, above the standard set by WHO. The study aimed to provide an accurate picture of the eating behavior of adolescent girls and its impact and to serve as a basis for developing future policies and strategies in achieving the SDGs 2030 targets related to nutrition.

## Materials and methods

This review follows the Preferred Reporting Items for Systematic Reviews and Meta-Analyses (PRISMA) guidelines (Moher et al., 2009). Additionally, it was registered in the International Prospective Register of Systematic Reviews database (PROSPERO) with registration number CRD42023389909.

### Search strategy

The search strategy was determined based on research questions as well as inclusion and exclusion criteria based on the PICOS (Participants, Intervention, Comparison, Outcomes, and Study design model) (Chandler et al., 2022) as shown in Table 1:

**TABLE 1:** Inclusion and exclusion criteria.

	Inclusion criteria	Exclusion criteria
Participant	Adolescent girls	Studies with participants other than adolescent girls, such as studies involving adult women, men, or children, will be excluded
Intervention	The systematic review did not focus on a specific intervention but aimed to evaluate the eating behavior of adolescent girls in countries with a high prevalence of stunting in young children	Since the review doesn’t focus on a specific intervention, there may not be specific exclusion criteria related to interventions
Comparison	There was no specific comparison group in this systematic review, as it primarily aimed to assess eating behavior in the target population without a direct comparison to another group	Studies that involve a direct comparison between different interventions or groups (e.g., an intervention group and a control group) may be excluded if the review’s focus is primarily descriptive and doesn’t involve comparing interventions
Outcome	The outcome measures included various aspects of eating behavior, dietary patterns, and related factors in adolescent girls, such as diet quality, meal skipping, dieting behaviors, body image perception, dietary diversity, and the presence of eating disorders	Studies that do not report relevant outcomes related to eating behavior, dietary patterns, body image, or related factors in adolescent girls will be excluded. Studies with outcomes unrelated to the research questions of the systematic review will also be excluded
Study Design	The review included studies with various study designs, primarily cross-sectional, as well as one mixed-method explanatory design. These studies aimed to investigate eating behavior in adolescent girls in countries with a high prevalence of stunting in young children	Studies with study designs that do not align with the objectives of the systematic review, such as animal studies, case reports, or studies without primary data (e.g., review articles), will be excluded
Language	All language	None
Setting	All setting	None

The search strategy used in PubMed for this systematic review is designed to identify relevant studies related to adolescent girls’ eating behavior and its association with stunting. The strategy includes the following components; Participants (P): MeSH Term: “adolescent” and Keywords in Title/Abstract: “girls,” Outcome (O): MeSH Term: “feeding behavior” and Keywords in Title/Abstract: N/A (MeSH term covers the outcome), Exposure (E): Keywords: “growth disorders,” “growth,” “stunting,” “stunted.”

The Boolean operators “AND” and “OR” are used to combine these components effectively. The search seeks to find studies that involve adolescents (using MeSH terms and title/abstract keywords), address eating behavior (using MeSH term), and explore the association with growth disorders, including stunting (using various keywords).

The search strategy aims to be comprehensive by including both MeSH terms and additional keywords to ensure that relevant studies are captured. This approach helps in identifying a wide range of articles that match the research focus of the systematic review.

The search started in the PubMed database in January 2023 (as seen in Table 2) and has been filtered since 2000. Later, it was replicated in three other databases: CINAHL, EMBASE, and Scopus. Then, two clusters of search terms were designed. The first term includes terms related to stunting, while the second relates to eating behavior. Finally, words were selected from the Medical Subject Headings (MeSH) thesaurus to develop an advanced representative search.

**TABLE 2:** Search strategy in PubMed.

Search strategy
Search: **(((adolescent[MeSH Terms]) AND (girls[Title/Abstract])) AND (behavior, eating[MeSH Terms])) AND (stunting)** “adolescent”[MeSH Terms] AND “girls”[Title/Abstract] AND “feeding behavior”[MeSH Terms] AND (“growth disorders”[MeSH Terms] OR (“growth”[All Fields] AND “disorders”[All Fields]) OR “growth disorders”[All Fields] OR “stunting”[All Fields] OR “stunted”[All Fields]) **Translations** **adolescent[MeSH Terms]:** “adolescent”[MeSH Terms] **behavior, eating[MeSH Terms]:** “feeding behavior”[MeSH Terms] **stunting:** “growth disorders”[MeSH Terms] OR (“growth”[All Fields] AND “disorders”[All Fields]) OR “growth disorders”[All Fields] OR “stunting”[All Fields] OR “stunted”[All Fields]

### Study selection process

This review was conducted in three main stages. The first stage was identifying and removing duplications. The second stage was selecting studies based on inclusion/exclusion criteria by reviewing article titles and abstracts. The final stage was reviewing the eligible articles by reading their full text. Two independent reviewers conducted all of these stages, and if there was a disagreement, a third reviewer was assigned to resolve it. The inter-reviewer agreement for the overall study selection was good, with a Cohen Kappa Index of 0.82 (95% CI, 0.49–1.00) (McHugh, 2012).

## Result

### Study selection

**FIGURE 1:**
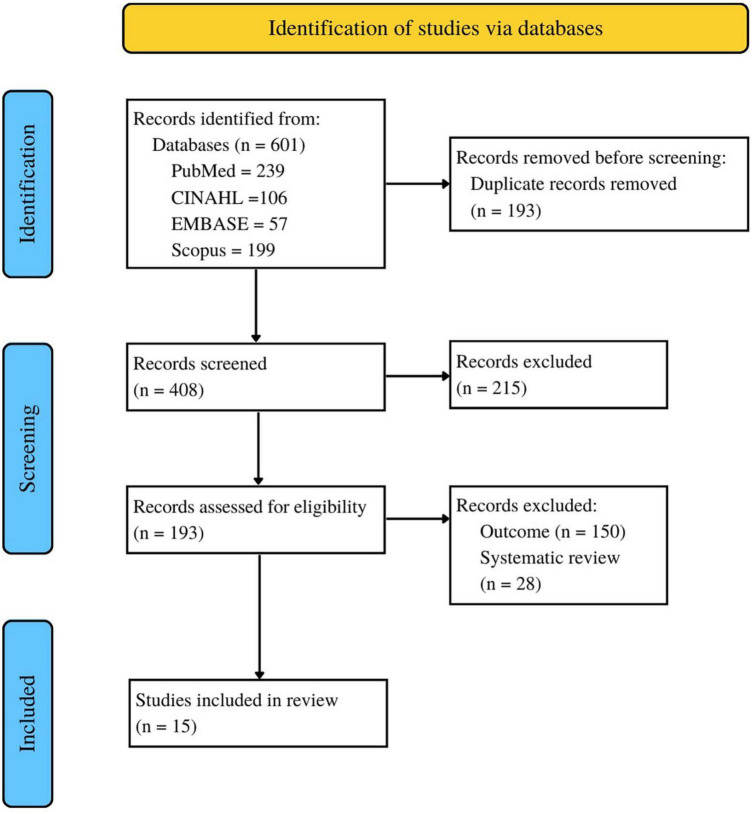
PRISMA flow diagram of the included and excluded studies throughout the systematic review protocol.

The search strategy resulted in a total of 601 potentially relevant studies. Of these, 408 studies met the inclusion criteria based on a review of the title and abstract. After reading the complete text, 393 articles were excluded because they did not meet the inclusion criteria, such as not being conducted in countries with a high prevalence of stunting or not focusing on adolescent girls. Finally, only 15 studies met the eligibility criteria and proceeded to the next stage. The screening process can be seen in Figure 1.

### Study characteristics

The variables reviewed in the selected studies have been described in Table 3:

**TABLE 3:** Study characteristics.

No	References/ Country	Design (study type)	Target population (Age)	Inclusion criteria	Exclusion criteria	Sample size	Assessment tool	Target eating behavior	Statistical analysis	Objective	Outcome	Result	Cite
1	Debasish et al., 2021/India	Explanatory mixed method design	Adolescent girls aged 10–19 years	All sampled adolescent girls	Pregnant and lactating adolescent girls	250	FGD with 32-item check list followed according to the consolidated Criteria for Reporting Qualitative Research	Practice diet, food choice	N-Vivo 8 for the qualitative data	To explore the perception of body image if it affected their eating behavior	Skip major meal on purpose to lose weight	7% of the adolescent girls did dieting for maintaining body shape.	Debasish L, Premarajan KC, Murugan V. Aesthetic conflict and eating behavior in adolescent girls of urban slum- A community based mixed method design. J Clin Diagnost Res. 2021; 15(4): LC20-4.
											Applying dietary restraint	They strove to achieve this goal by applying dietary restraints. The major response in this regard was that they wanted to be either thin or normal in weight but did not want to be fat by any means.	
											Avoided taking any solid food	As food was attributed to make one fat or thin, some adolescent girls skipped major meals to become slim or avoided taking any solid food (especially rice) for liquid diet (juice or milk). As told by a respondent,	
2	Mishra and Mukhopadhyay, 2011/India	Cross-sectional study	Adolescent girls aged 15–19 years (married and unmarried)	All sampled adolescent girls	None	577	Food Frequency Questionnaire	Food habits, dietary behaviors	Factor analysis and varimax rotation	To evaluates the prevalence of weight concerns and subsequent eating behavior modifications among a group of adolescent girls in Sikkim.	Food categorized diet	Younger adolescents (<18 years) had higher scores for ‘snacks–ice cream–beverages’ compared to older girls (≥18 years)	Mishra SK, Mukhopadhyay S. Eating and weight concerns among Sikkimese adolescent girls and their biocultural correlates: an exploratory study. Public Health Nutrition. Cambridge University Press; 2011;14(5):853–9.
											Weight reduction diet	Urban girls tend to be on a weight by skipped their meals or consumed snacks in place of normal meals	
3	Dixit et al., 2014/India	Cross-sectional study	Adolescent girls age 10–19 years	All sampled adolescent girls	None	576 adolescent girls aged 10–19 years	A self-designed pretested schedule questionnaire	Eating behavior, nutrient intake evaluation	Descriptive analysis	To study the specific trends and transitions in the eating behavior of adolescent girls of Lucknow, residing in rural, urban, and slum localities and its correlation with their nutrient intake and nutritional status.	Dietary habits type	The three types of dietary habits identified were non-vegetarian (40.8%), vegetarian (35.5%), and eggetarian (23.7%). Poor eating behavior was more prevalent in rural areas (11.9%) compared to urban and slum areas. Stunting was found to be significantly associated with the eating behavior of adolescent girls	Dixit S, Singh JV, Kant S, Agarwal GG, Dubey A, Kumari N. A cross-sectional study on predictors and significance of eating behavior of adolescent girls. Vulnerable Children and Youth Studies [Internet]. 2014 Jan [cited 2023 Jan 7];9(1):10–6.
4	Soo et al., 2008/Malaysia	Cross-sectional study	Adolescents girls grade 10–11 of national secondary school	All sampled adolescent girls	Drop outs	489 adolescent girls aged 15–16 years	Restrained eating scale of the Dutch Eating Behavior Questionnaire	Dietary restraint	Pearson product-moment correlation	To examine disordered eating behaviors of adolescent girls in Malaysia and to estimate associations with body weight, body-size discrepancy, and self-esteem	Restrained Eating	Restrained eating was significantly correlated with Body Mass Index (*r* = 0.34, *p* < 0.001) and Body-size Dissatisfaction (*r* = 0.21, *p* < 0.001).	Kah Leng Soo, Zalilah Mohd Shariff, Mohd Nasir Mohd Taib, Bahaman Abu Samah. Eating Behavior, Body Image, and Self-Esteem of Adolescent Girls in Malaysia. Perceptual and Motor Skills [Internet]. 2008 Jun [cited 2023 Jan 7];106(3):833–44
5	Mahriani et al., 2022/Indonesia	Cross-sectional study	Adolescent girls aged 10–15 years.	Middle school girls who met the following criteria: (i) were in the age range of 10–15 years, (ii) had no chronic systemic disease, and (iii) were willing to participate in the study (proofed by parental signed informed consent).	None	96 Adolescent girls aged 12–15 years.	Adolescent Food Habit Checklist Index questionnaire	Eating behavior	Descriptive analysis	To analyze the relationship between diet assessment and oral health status of adolescent girls, the relationship between oral hygiene behavior and oral health status of adolescent girls, and the simultaneous relationship between dietary assessment and oral hygiene behavior with the oral health status of adolescent girls	Craving induced diet	From the data of 96 teenage girls, it shows that there is eating behavior based on the girls’ cravings. The girls start to eat anything as long as they feel full.	Mahriani Y, Indriyanti R, Musnamirwan IA, Setiawan AS. A cross-sectional study on dietary assessment, oral hygiene behavior, and oral health status of adolescent girls. Front Nutr. 2022 Oct 5;9:973241
6	Worku et al., 2022/Ethiopia	Cross-sectional study	All school girls attending high schools in Yeka sub-city	Adolescent girls attending school at selected schools during data collection.	Adolescent girls who did not come to school during data collection	265 adolescent girls	Household food secure	Dietary diversity	Descriptive analysis, logistic regression	To assess the magnitude of dietary diversity and associated factors among high school adolescent girls at the selected school of Yeka Sub-city, Addis Ababa, Ethiopia 2021.	Individual dietary diversity score and household food insecure access scale	The mean dietary diversity score was 4.9 ± 1.47, with a high response rate of 97.2% among the proposed number of participants. Results showed that 56.7% of the adolescents had high dietary diversity scores, while 43.3% had low scores. Several factors were identified to be statistically associated with low dietary diversity scores. These factors included spending a long time on social media (adjusted odds ratio = 2.6), school type (adjusted odds ratio = 6.5), educational status of mother (adjusted odds ratio = 8.7), consuming more sweet food (adjusted odds ratio = 3.6), occupation of the mother (adjusted odds ratio = 2.3), household security (adjusted odds ratio = 2.3), and fear of being obese or worried about shape (adjusted odds ratio = 5.0). These findings suggest that a variety of social, economic, and psychological factors may play a role in shaping the dietary habits of adolescents. Overall, the study highlights the need for promoting healthy dietary habits among adolescents, particularly those who are at risk of having low dietary diversity scores.	Worku L, Mamo K, Bekele T, Atlaw D. Dietary diversity score and associated factors among high school adolescent girls in a selected school of Yeka Sub-city, Addis Ababa. SAGE Open Med. 2022 Apr 26;10:20503121 221094896.
7	Visser et al., 2014/South Africa	Cross-sectional study	Adolescent girls grade 8–12	Current learner at the school of study. Self-identified follower or guardian written consent displayed good comprehension of the English language	None	261 adolescent girls	Eating Attitudes Test (EAT-26)	Risk eating behavior	*T*-test to compare means	This study aimed to determine the prevalence of abnormal eating attitudes and weight-loss behavior in female Jewish adolescents. Teachers’ awareness of these factors and their attitudes toward a school programme to address these were also investigated.	Eating Attitudes	Twenty percent of the participant had scored ≥ 20 on the EAT-26, which is suggestive of a possible eating disorder. Surprisingly, currently trying to lose weight was not selected, possibly because the finding was so common in the study population.	J. Visser BSc Dietetics, M Nutrition; Senior Lecturer, T. Notelovitz MBBCh, MNutrition; Private Practice, Cp Szabo MBBCh, FCPsych, MMed, PhD, MSc (Med), Professor and Head and N. Fredericks BSc (Dietetics), MSc (Nutrition Management) (2014) Abnormal eating attitudes and weight-loss behavior of adolescent girls attending a “traditional” Jewish high school in Johannesburg, South Africa, South African Journal of Clinical Nutrition, 27:4, 208-2016. doi: 10.1080/16070658.2014.1173451
8	Kurschner et al., 2020/Guatemala	Qualitative Cross-sectional study	Adolescent girls 15–19 years old			24	Interview	Dietary habits	Qualitative analysis	To address the impact of out-of-school status on diet and physical activity	Dietary habits type	Irregular/disrupted eating schedule	Kurschner S, Madrigal L, Chacon V, Barnoya J, Rohloff P. Impact of school and work status on diet and physical activity in rural Guatemalan adolescent girls: a qualitative study. Ann N Y Acad Sci. 2020 May;1468(1):16–24. doi: 10.1111/nyas.1418. Epub 2019 Jul 30. PMID:31361343; PMCID: PMC7317776.
9	Alam et al., 2010/Bangladesh	Cross-sectional study	Adolescent 13–18 years old	Never married adolescent		4.993	Questionnaire asked by interview method	Dietary pattern	Bivariate and logistic regression	This study estimated the levels and differentials in nutritional status and dietary intake and relevant knowl- edge of adolescent girls in rural Bangladesh using data from the Baseline Survey 2004 of the National Nutrition Programme		Consumption of staple food (rice or wheat) in the last 7 days was universal with no difference be- tween the asset quintiles. Consumptions of non-staple food items, such as meat, eggs, *dal (*lentils), fruits, and leafy vegetables, were not frequent in rural areas. Half of them did not eat meat and milk each, and 40% did not eat eggs at all. The average days of consumption in a week were 3.4 for fish, 0.6 for meat, 1.1 for eggs, 1.9 for milk, 1.7 for *dal* and leafy vegetables each, and 2.3 for fruits. The availability and consumption of fruits is quite seasonal in rural Bangladesh	Alam N, Roy SK, Ahmed T, Ahmed AM. Nutritional status, dietary intake, and relevant knowledge of adolescent girls in rural Bangladesh. J Health Popul Nutr. 2010 Feb;28(1):86–94. doi: 10.3329/jhpn.v28i1.452. PMID:20214090; PMCID: PMC2975850.
10	Tengia-Kessy and Killenga, 2020/Tanzania	Cross-sectional study	Adolescent girls age 10–19 years.			427 adolescent girls	Food Frequency questionnaire	Dietary habits	Descriptive analysis	This study hence assessed the magnitude and correlates of EBW among secondary school adolescent girls in order to inform stakeholders developing strategies for mitigating overweight and obesity among this population sub-group.	Dietary habits	the proportion of adolescents with excess body weight (BMI > + 1SD) was 23%. The majority (63%), reported unhealthy dietary habits while half (51.5%) of them had moderate level of knowledge on healthy eating. Compared to working as a civil servant, the odds of having excess body weight among girls whose mothers/female guardians were housewives was less by 60% (aOR = 0.4, 95% CI: 0.2, 0.9). Furthermore, the odds of having excess body weight among adolescents eating unhealthy foods were almost six times higher compared to their peers on healthy diet (aOR = 5.8, 95% CI: 2.9, 11.3)	Tengia-Kessy A, Killenga JN. Prevalence of excess body weight and associated factors among secondary school adolescent girls in northern Tanzania: a cross-sectional study. Pan Afr Med J. 2020 Nov 19;37:253. doi: 10.11604/pamj.2020.37.253.2534. PMID:33598068; PMCID: PMC7864264.
11	Agofure et al., 2021/Nigeria	Cross-sectional study	adolescent female students (12–18 years old)	N.a	N.a	201 adolescent female students	Questionnaire	Dietary pattern	Descriptive statistics	This study was designed to investigate the dietary patterns and nutritional status of the female adolescents in Amai Secondary Commercial School, Delta State, Nigeria	Unhealthy dietary pattern	Less than half of the respondents 83 (41.30%) affirmed that they eat eggs sometimes while 67 (33.30%) affirmed that they take milk sometimes and 71 (35.30%) affirmed that they eat sweet sometimes. Also, less than half 91 (45.30%) confirm that they take ice cream sometimes while 78 (38.80%) of the respondents agreed that they take fruits sometimes and almost half 96 (47.80%) confirmed that they take garri/eba very often. In addition 69 (34.40%) of the respondents observe three square meals sometimes while 77 (38.30%) established that they take breakfast very often and almost half of the respondents 97 (48.30%) confirm that they eat fruits after meals sometimes	Otovwe Agofure et al. Dietary pattern and nutritional status of female adolescents in Amai Secondary School, Delta State, Nigeria. Pan African Medical Journal. 2021;38(32). doi: 10.11604/pamj.2021.38.32.15824
12	Birru et al., 2018/Ethiopia	Cross-sectional study	Adolescent girls attending both private and governmental	All girls attending the survey period		768 adolescent girls	Questionnaire	Dietary diversity	Binary logistic regression model	This study assessed dietary and associated factors among school adolescent girls in Gondar city, northwest Ethiopia	Dietary diversity	75.4% adolescent girls had adequate dietary diversity. Moreover, the mean dietary diversity score of participants was 5.76 ± 1.81. Majority (97. 7%) of adolescent girls consumed starchy staples (grains, roots and tuber). However, only 32.4% ate fruits	Birru SM, Tariku A, Belew AK. Improved dietary diversity of school adolescent girls in the context of urban Northwest Ethiopia: 2017. Ital J Pediatr. 2018 Apr 25;44(1):48. doi: 10.1186/s13052-018-0490. PMID:29695264; PMCID: PMC5918967.
13	Berheto et al., 2015/Ethiopia	Cross-sectional study	Adolescent girls from rural and urban grades 5 - 10			622 subjects	A four-item individual food security scale	Dietary pattern	Descriptive analysis	This study was therefore performed to help redress this lack of data and to provide information for future improvements by health planners and policy makers.	Dietary pattern	There were no significant differences in the ages or physical activities of the two populations of girls studied. Consumption of cereal, vegetables, sweets, sugars, fats, meat, and eggs was similar between the two groups, although slight differences were found with regard to legumes, milk, and fruit consumption.	Berheto TM, Mikitie WK, Argaw A. Urban-rural disparities in the nutritional status of school adolescent girls in the Mizan district, south-western Ethiopia. Rural Remote Health. 2015 Jul-Sep;15(3):3012. Epub 2015 Jul 30. PMID:26235698.
14	Javaid et al., 2020/Pakistan	Cross-sectional study	Adolescent girls age 10–14 years	Adolescent from female government schools		385 female students	Open ended questionnaire	Eating habits	Descriptive analysis	The objective of this study was to know the determinants of eating habits and physical activities among school-going female adolescents of public sectors schools of Rawalpindi Pakistan.	Eating habits	Mostly (41.9%) students in this study were found obese and (9.2%) were malnourished. Many students (38.6%) students said that they eat confectionary like; chocolates and toffees in school. More than half of the respondents (52.6%) eat the vegetables rarely in their diet. (54.4%) were those who take fruits daily in their diet and (41.1%) said that they eat meat in their diet rarely. (61.7%) respondents reported that they weekly. Above half (58.6%) were those students who eat deep fried potato chips daily. Nearly half of the students were eating snakes (48.2%), cakes (47.7%) and chocolate (88.8%) in their daily diet in spite majority (93%) of them knows that the junk food is not good for health. Mainly students (83.4%) has reported that they are using milk less than one cup in their diet daily and two thirds (66.7%) students intake water more than five cups every day take meat product in diet	Javaid F, Sheerani NL, Haider S, Anwar F, Azad M, Kumari P, Kanwal S, Kumar R. Determinants Of Eating Habits And Physical Activity Among Female Students Of Government Schools Of Urban City Of Pakistan. J Ayub Med Coll Abbottabad. 2020 Oct-Dec;32(4):517–522. PMID:33225655.
15	Maria Soares and Yamarat, 2014/Timor Leste	Cross-sectional study	Adolescent girls aged 14–19 years old	Female 14–19 years old, willing to participate in study and exclusion criteria those who are not present at the time of data collection		244	Self-administration with structured questionnaire	Food consumption habits	Chi-square test	The objective of the study was to determine the host, agent, and environment factors associate with nutritional status of adolescent girls aged 14–19 years old in three secondary high schools of Manatuto district, Timor-Leste	Food consumption habits	All most (59.9%) of respondents had low calories intake < 2,200 Kcal per person per day and all most (61.8%) of respondent had low protein gram intake less than 50 gram based on protein gram requirements for aged 13–19 years old (per person per day). Majority of respondent reported (84.4%) no eating liver during a week, 43.9–63.1% eating red meat, fish, egg and chicken 1–2 times per week. About 49.1–54.9% mustard, white pumpkin and potato leaf 1–2 times per week. Majority 48.8–64.8% respondents reported eating mango, tomato, pineapple, guava, papaya and avocado 1–2 times per week. Majority 57.8% of respondents reported eating red bean 1–2 times per week, 11.9% eating red bean 3–5 times per week and only 9.0% eating 6–7 times per week. Haft of respondents 59.0% reported eating rice 5–6 times per week, 53.3% respondents eating corn, 61.3% of them eating Cassava 1–2 per week. Of 49.6–57.8% respondents reported eating taro, sweet potato, potato and noodle 1–2 times. Nearly haft of respondents 31.1–49.5% reported drinking milk with coffee, coffee, soft drink, tea, and milk 1–2 respondents reported eating spinach, cassava leaf, pumpkin leaf, times per week.	Soares, D. M., and Yamarat, K. (2017). Nutritional Status of Secondary High School Adolescent Girls Ages 14–19 Years, Manatutuo District, Timor-Leste. *Journal of Health Research*, *28*(4), 241–248.

#### Country and design of the studies

Most of the studies in this review utilized a cross-sectional design, with only one using a mixed-method explanatory design (Debasish et al., 2021). The studies were conducted in India (Mishra and Mukhopadhyay, 2011; Dixit et al., 2014; Debasish et al., 2021), Malaysia (Soo et al., 2008), Indonesia (Mahriani et al., 2022), Ethiopia (Berheto et al., 2015; Birru et al., 2018; Worku et al., 2022), South Africa (Visser et al., 2014), Guatemala (Kurschner et al., 2020), Bangladesh (Alam et al., 2010), Tanzania (Tengia-Kessy and Killenga, 2020), Nigeria (Agofure et al., 2021), Pakistan (Javaid et al., 2020), and Timor Leste (Maria Soares and Yamarat, 2014).

#### Target population

Although the target population of this study is adolescent girls, not all studies focus on the same age range, the adolescent period is divided into three stages: early (ages 11–14), mid (ages 15–17), and late (ages 18–21) (Mahriani et al., 2022). In this study, two studies only focused on the early stage (Visser et al., 2014; Javaid et al., 2020), one study on the mid-stage (Soo et al., 2008), three studies on the early to mid-stage (Maria Soares and Yamarat, 2014; Berheto et al., 2015; Mahriani et al., 2022), two studies on the mid to late-stage (Mishra and Mukhopadhyay, 2011; Kurschner et al., 2020), and the majority of studies focused on the entire adolescent period from early to late (Alam et al., 2010; Dixit et al., 2014; Birru et al., 2018; Tengia-Kessy and Killenga, 2020; Agofure et al., 2021; Debasish et al., 2021; Worku et al., 2022). These studies are listed with the age range mentioned in the text, along with the study number and corresponding reference.

#### Sample size

There is a difference in sample size among these studies, ranging from 24 subjects in the qualitative design study 20 to 4,993 subjects in the sizeable community-based study in Bangladesh. Therefore, this systematic review evaluated 9,831 participants.

#### Assessment tools

All studies utilize different assessment tools to evaluate changes in eating behavior targets. Four studies employed self-designed questionnaires (Dixit et al., 2014; Maria Soares and Yamarat, 2014; Birru et al., 2018; Agofure et al., 2021), while the others used standardized, validated scales such as the Food Frequency Questionnaire (Mishra and Mukhopadhyay, 2011; Tengia-Kessy and Killenga, 2020), Dutch Eating Behavior Questionnaire (Soo et al., 2008), Adolescent Food Habit Checklist (Mahriani et al., 2022), Household Food Security (Berheto et al., 2015; Worku et al., 2022), and Eating Attitude Test (Visser et al., 2014). Qualitative research employs assessment tools in the form of open-ended questionnaires delivered through focus group discussions (FGD) (Debasish et al., 2021) and interviews (Alam et al., 2010; Javaid et al., 2020; Kurschner et al., 2020).

#### Objective, outcomes, and result

Table 3 also displays this systematic review’s objective, outcome, and result. All studies have a similar objective: to assess eating/dietary behavior in adolescent girls. Four studies link diet habits to the activities of adolescent girls in school (Maria Soares and Yamarat, 2014; Birru et al., 2018; Javaid et al., 2020; Kurschner et al., 2020), two studies are related to body image perception (Mishra and Mukhopadhyay, 2011; Debasish et al., 2021), one study examines the transition of eating behavior in urban, rural, and slum areas (Dixit et al., 2014), two studies assess eating disorders directly in adolescent girls (Soo et al., 2008; Visser et al., 2014), three studies examine dietary diversity, patterns, or habits (Alam et al., 2010; Tengia-Kessy and Killenga, 2020; Mahriani et al., 2022; Worku et al., 2022), and the last study aims to provide data information to policymakers or planners (Berheto et al., 2015). The outcomes of this study can be summarized as dietary restraint, unhealthy dietary patterns, dietary types, and eating behavior in adolescent girls.

The results of the studies generally show the presence of various eating behaviors in adolescent girls in the countries where the studies were conducted. Adolescent girls who diet to maintain body shape may avoid or skip meals to remain thin (Mishra and Mukhopadhyay, 2011; Javaid et al., 2020; Debasish et al., 2021; Worku et al., 2022), especially in urban areas (Mishra and Mukhopadhyay, 2011). However, poor eating behavior was also found among adolescent girls in rural areas, potentially leading to stunting (Dixit et al., 2014). There is also a tendency among adolescent girls to eat only when they feel hungry (Mahriani et al., 2022). Unfortunately, unhealthy dietary habits associated with excess body weight can lead to the consumption of unhealthy foods (Tengia-Kessy and Killenga, 2020). A study also reported a healthy eating pattern of three meals a day, but the meals were not well-balanced (Agofure et al., 2021). For example, in a study conducted in Timor Leste, adolescent girls were found to consume insufficient calories and protein in their meals (Maria Soares and Yamarat, 2014).

Eating behavior is also related to Dietary Diversity (DD), which refers to consuming various food groups or types within a specific period. Consuming diverse foods is advantageous for obtaining a variety of macro and micronutrients and is essential for ensuring sufficient nutrient intake (Weerasekara et al., 2020). The adolescent girls in this reviewed study were found to have low dietary diversity, associated with several factors, including spending too much time on social media, consuming more sweet foods, and again, fear of gaining weight (Worku et al., 2022).

Irregular or disrupted eating schedules can also factor in developing unhealthy eating behaviors. Busy school schedules often cause adolescent girls to skip meals or rush through them, leading to loss of appetite or more meal skipping and irregular snacking (Kurschner et al., 2020). Inadequate dietary diversity was found in adolescent girls who consume few fruits and a large number of starchy staples (Birru et al., 2018). The type of food also became the focus of the review study. Adolescent girls in Ethiopia more frequently consume staple foods such as rice or wheat than non-staple foods such as eggs, meat, and fish. Fruits are more commonly consumed according to their seasonal availability (Alam et al., 2010; Berheto et al., 2015). Possible eating disorders were reported in one study (Visser et al., 2014).

### Quality of studies

The Newcastle-Ottawa Quality Assessment Scale (NOS) (Stang, 2010; Lo et al., 2014) was utilized to evaluate the quality of the studies. This tool was designed to aid systematic reviews in reaching quality criteria while minimizing bias risks. The NOS comprises three main categories: selection, comparability, and outcome, each consisting of several items about the features of observational studies, with several answer options. At least one answer within each category is accompanied by a star (6) which signifies a low risk of bias. Upon completion of the scale, the stars are tallied, and if the score is less than seven stars, there may be a high risk of bias. Additionally, if the reviewers select answers without stars, it is important to examine possible study biases. NOS scores are categorized into three groups: very high risk of bias (0–3 stars), high risk of bias (4–6 stars), and low risk of bias (7–9 stars) (Lo et al., 2014).

Two independent reviewers assessed the quality of each study, with a third reviewer assigned in case of disagreements. The final consensus is presented in Table 4. The Cohen Kappa Index was used to measure the level of agreement among reviewers in assessing the overall quality of the studies. The findings indicated a strong agreement (Cohen Kappa Index = 0.76; 95% CI, 0.60–0.89) in evaluating the individual scores of each study. Based on this analysis, it can be inferred that all fifteen studies had a minimal risk of bias.

**TABLE 4:** Quality appraisal study.

References	Selection			Comparability			Outcome		Total	Score
	**Representativeness of the exposed cohort**	**Selection of the non-exposed cohort**	**Ascertainment of exposure**	**Demonstration that the outcome of interest was not present at the start of the study**	**Comparability of cohorts on the basis of the design or analysis**	**Assessment of outcome**	**Was follow-up long enough** **for outcomes to occur**	**Adequacy of the follow-up**		
Debasish et al., 2021/India	✓	✓	✓	✓	✓	✓	✓	✓	8	Low risk of bias
Mishra and Mukhopadhyay, 2011/India	✓	✓	✓	✓	✓	✓			7	Low risk of bias
Dixit et al., 2014/India	✓	✓	✓	✓	✓	✓	✓	✓	8	Low risk of bias
Soo et al., 2008/Malaysia	✓	✓	✓	✓	✓	✓	✓	✓	8	Low risk of bias
Mahriani et al., 2022/Indonesia	✓	✓	✓	✓	✓		✓	✓	8	Low risk of bias
Worku et al., 2022/Ethiopia	✓	✓		✓	✓	✓	✓	✓	7	Low risk of bias
Visser et al., 2014/South Africa	✓	✓	✓	✓	✓	✓	✓		7	Low risk of bias
Kurschner et al., 2020/Guatemala	✓	✓	✓	✓	✓	✓	✓	✓	8	Low risk of bias
Alam et al., 2010/Bangladesh	✓	✓		✓	✓	✓	✓	✓	7	Low risk of bias
Tengia-Kessy and Killenga, 2020/Tanzania	✓	✓	✓	✓	✓	✓	✓		7	Low risk of bias
Agofure et al., 2021/Nigeria	✓	✓	✓	✓	✓	✓	✓	✓	8	Low risk of bias
Birru et al., 2018/Ethiopia	✓	✓	✓	✓	✓	✓	✓	✓	8	Low risk of bias
Berheto et al., 2015/Ethiopia	✓	✓	✓	✓	✓	✓	✓		7	Low risk of bias
Javaid et al., 2020/Pakistan	✓	✓	✓	✓	✓	✓	✓	✓	8	Low risk of bias
Maria Soares and Yamarat, 2014/Timor Leste	✓	✓	✓	✓	✓	✓	✓	✓	8	Low risk of bias

## Discussion

During adolescence, there is a need for greater nutritional intake to support rapid growth and ensure a healthy lifestyle for teenagers. An additional study indicated that teenagers’ choices regarding their lifestyle and diet could either negatively affect their health during this transition period or contribute positively to their overall wellbeing as they become adults (Amer and Kateeb, 2021). In this systematic review involving fifteen studies, a comprehensive overview of eating behavior in adolescent girls in countries with high stunting prevalence was obtained. The observed eating behaviors from this systematic review include eating disorder (Soo et al., 2008; Visser et al., 2014), weight reduction diet (Mishra and Mukhopadhyay, 2011; Debasish et al., 2021), restrained eating (Soo et al., 2008; Alam et al., 2010; Kurschner et al., 2020; Tengia-Kessy and Killenga, 2020; Agofure et al., 2021), craving-induced eating (Mahriani et al., 2022), unhealthy dietary pattern (Agofure et al., 2021), and low diversity diet (Birru et al., 2018; Worku et al., 2022).

## Relationship between eating disorders and extreme weight-loss behaviors

The first crucial finding of this systematic review underscores the connection between eating disorders and extreme weight-loss behaviors among adolescent girls. Only four per cent of the surveyed teenage girls were diagnosed with an eating disorder using the EAT-26 measurement tool. Notably, individuals who engaged in extreme weight-loss behaviors in the past year were likelier to score higher on the EAT-26, indicating a potential risk of eating disorders (Visser et al., 2014). These results emphasize the need for early identification and support for individuals at risk for or already experiencing disordered eating patterns. Psychological, genetic, environmental, and cultural factors may contribute to the development of eating disorders (Loth et al., 2014).

Additionally, the study found that perceiving oneself as overweight was a predictor of higher total scores on the EAT-26 and a score equal to or greater than 20, often used as a cut-off point for identifying individuals at risk for an eating disorder. This finding aligns with previous research showing a correlation between perceiving oneself as overweight and experiencing eating problems during adolescence. It also suggests that individuals who perceive themselves as overweight may be more likely to engage in weight-loss behaviors (Mateos-Padorno et al., 2010). These findings highlight the importance of recognizing the relationship between extreme weight-loss behaviors, body perception, and the risk of developing an eating disorder. Identifying individuals who exhibit these behaviors and perceptions can help in early intervention and support for individuals at risk for or already experiencing disordered eating patterns (Visser et al., 2014).

## Body perception and risk of eating disorders

Our second key finding revolves around body perception and its role in the risk of developing eating disorders among adolescent girls. Notably, perceiving oneself as overweight predicted higher total scores on the EAT-26. This aligns with prior research showing a correlation between perceiving oneself as overweight and engaging in extreme weight-loss behaviors. Recognizing this relationship is crucial for targeted interventions and support.

Adolescent girls commonly experience dissatisfaction with their body image and negative self-perception, affecting their eating behavior. In the study we reviewed, most participants reported skipping at least one meal per day. Among this group, some intentionally skipped meals to lose weight, commonly called dieting. When comparing the practice of dieting among all the reasons for skipping meals with the participants’ self-perception of their body shape, it was observed that those who engaged in dieting were only individuals who perceived their body shape as fat or normal, while none of those who considered themselves as thin reported dieting (Debasish et al., 2021).

## Influence of body image on dieting behaviors

In our third finding, we delve into body image’s influence on adolescent girls’ dieting behaviors. It was observed that those who engaged in dieting were exclusively individuals who perceived their body shape as fat or normal, while none of those who considered themselves thin reported dieting. This underscores the complexity of body image perceptions and their impact on adolescent dietary choices.

Initially, the process of perceiving body image is believed to be narrow. Still, over time it can develop into body distortion, where there is a significant alteration in the perception of body size. This means that individuals may perceive themselves as fat even when dangerously underweight (Farid and Kamrani, 2016). This tendency to desire thinness was also observed quantitatively in the current study. There was a lack of agreement between adolescents’ perceptions of themselves and their actual body size. Therefore, it became evident that their beliefs about their bodies were based on a mistaken notion. It was concerning that those girls who perceived themselves as normal or fat in the present study were underweight. A study conducted in Korea on adolescents (Su-Jung and Jong-Ho, 2021) found that underweight or normal-weight individuals who perceived themselves as overweight were at a higher risk of developing eating disorders such as anorexia nervosa.

## Restrained eating behavior

Our fourth important finding highlights restrained eating (RE) behaviors among young adolescents. This vulnerability is linked to increased weight concerns, body dissatisfaction, and problematic eating behaviors. Adolescents who exhibited RE behaviors tended to eat only when hungry or craving, which contradicts previous notions. Understanding the factors contributing to craving-induced diet behavior, such as limited access to food at school, is critical for developing effective interventions.

The restrained eating (RE) identified in four reviewed studies (Soo et al., 2008; Alam et al., 2010; Kurschner et al., 2020; Tengia-Kessy and Killenga, 2020; Agofure et al., 2021) indicates that young adolescents are especially susceptible to RE. This vulnerability stems from their increased likelihood of experiencing weight concerns, body dissatisfaction, and problematic eating behaviors. The restrained eating behavior exhibited by adolescents in Mahriani et al.’s (2022) study involves only eating when hungry or craving, contradicting findings that suggest adolescents frequently experience hunger (Kral et al., 2010; Savard et al., 2022). However, Mahriani et al.’s (2022) study reveals that the craving-induced diet behavior is primarily attributed to adolescents’ lack of knowledge regarding the benefits of proper nutrition and the difficulty in accessing food while they are at school.

## Unhealthy eating patterns during adolescence

Our fifth finding sheds light on the unhealthy eating patterns prevalent during adolescence, characterized by skipped meals, increased consumption of processed and fast foods, and decreased intake of fruits and vegetables. Despite knowledge about the importance of a balanced diet, adolescents frequently indulged in junk food, indicating that factors beyond knowledge influence their dietary choices.

During adolescence, characterized by rapid growth, individuals experience significant physical, psychological, hormonal, cognitive, and social changes. These transformations lead to fluctuations in the nutritional requirements, eating habits, and food preferences of the body. The eating pattern of adolescents differs from that of children. The higher academic and socio-economic pressures associated with this growth stage often result in skipped meals, an increased intake of processed and fast foods, and a decrease in the consumption of fruits and vegetables (Onyiriuka et al., 2013). The unhealthy eating patterns identified in the reviewed study indicate that adolescents tend skipped meals or to have inadequate consumption of vegetables and fruits while frequently indulging in junk food (Agofure et al., 2021). Furthermore, despite knowing the importance of a balanced diet for maintaining a healthy life, this knowledge did not reflect in the nutritional status of the respondents. This suggests that factors other than knowledge influence the dietary choices of adolescent girls (Ogunkunle and Oludele, 2013).

## Low-diversity diet and micronutrient deficiencies

The last crucial finding pertains to the presence of a low-diversity diet among adolescent girls, reflected in sub-optimal dietary intake and micronutrient deficiencies (Birru et al., 2018; Worku et al., 2022). This sub-optimal nutrition during adolescence can lead to delayed puberty, contracted pelvis, and unfavorable birth outcomes, including stunted growth in newborns. Recognizing the importance of dietary diversity is essential for addressing these nutritional challenges.

The dietary diversity score (DDS) is a measure that counts the number of different food groups consumed over a specific period (Davis, 2016). It reflects the diet quality at the household or individual level. Optimal nutrition is crucial during adolescence since this period is responsible for gaining 50% of adult weight, 20% of adult height, and 50% of skeletal mass (Hadush et al., 2021). However, research indicates that 45–60% of adolescent girls have sub-optimal dietary intake (Shashikantha et al., 2016), leading to various micronutrient deficiencies, including Vitamin A, iron, and iodine (Birru et al., 2018). Similar findings were observed in Ethiopia, where 29 and 30% of adolescent girls had thinness and anemia, respectively (Central Statistical Agency Ethiopia, 2016). These nutritional deficiencies can result in delayed puberty, contracted pelvis, and unfavorable birth outcomes, including stunted growth in newborns (Setiawan et al., 2022).

While this systematic review provides valuable insights into eating behaviors among adolescent girls in countries with high stunting prevalence, it is essential to acknowledge its limitations:

Limited Generalizability: The findings of this review are based on studies conducted in specific countries or regions with high stunting prevalence. As such, the generalizability of the results to a broader global context may be limited. Cultural, socioeconomic, and environmental factors that influence eating behaviors can vary significantly between regions, potentially affecting the applicability of these findings elsewhere.

Variability in Study Designs: The included studies in this review may have employed diverse methodologies, assessment tools, and criteria for measuring eating behaviors. This variability in study designs could introduce heterogeneity into the review, making it challenging to draw uniform conclusions.

Language Bias: The review’s search strategy may have been limited to studies published in English, which could introduce language bias. Relevant studies published in other languages may have been excluded, potentially affecting the comprehensiveness of the review.

Publication Bias: There is a possibility of publication bias, where studies with statistically significant or more sensational findings are more likely to be published. This bias could skew the review’s findings, potentially overemphasizing certain aspects of eating behaviors among adolescent girls.

Cross-Sectional Nature of Studies: Many of the included studies may have adopted cross-sectional designs, which provide a snapshot of eating behaviors at a specific point in time. Cross-sectional studies are limited in their ability to establish causality or track changes in eating behaviors over time.

Self-Reported Data: Several studies may have relied on self-reported data from adolescents. Self-reporting can introduce recall and social desirability biases, as participants may underreport or overreport certain behaviors due to social or cultural pressures.

Lack of Longitudinal Data: Longitudinal data that track eating behaviors and their consequences over an extended period are crucial for understanding trends and potential long-term effects. The absence of such longitudinal data may limit the review’s ability to explore the persistence and impact of certain eating behaviors.

Limited Focus on Interventions: This review primarily describes eating behaviors rather than evaluating interventions or prevention programs. While identifying eating behaviors is valuable, a more comprehensive review might explore the effectiveness of interventions in addressing these behaviors.

Evolving Nature of the Field: The field of nutrition and eating behaviors continually evolves. The review’s findings are based on studies available up to a specific date, with the most recent sources cited in this systematic review published in 2022 (Mahriani et al., 2022). Since then, new research may have emerged that could provide additional insights or alter the conclusions.

Potential Biases in Included Studies: The individual studies in the review may have biases and limitations, which can propagate to the review itself. The quality and rigor of the original studies can impact the overall strength of the review’s conclusions.

## Conclusion

In conclusion, the eating behaviors of adolescent girls in countries with a high prevalence of stunting exhibit concerning patterns, including a low diversity diet, sub-optimal dietary intake, and the development of various micronutrient deficiencies. These challenges are compounded by eating disorders, such as retained and craving-induced diet, which further impact their nutritional status. These eating disorders contribute to an unhealthy relationship with food and can harm their physical and mental wellbeing. Addressing these multifaceted issues requires a comprehensive approach encompassing nutritional education, promoting balanced diets, addressing the underlying factors contributing to eating disorders, and providing appropriate support and intervention for those affected. By addressing these eating behaviors and disorders, we can strive to improve adolescent girls’ overall health and wellbeing in these countries and reduce the prevalence of stunting and its associated health consequence.

## Data availability statement

The original contributions presented in this study are included in this article/supplementary material, further inquiries can be directed to the corresponding author.

## Author contributions

AS devised the project, the main conceptual ideas, the proof outline, composed the initial draft of the manuscript, and finalized the writing. AS and AB worked out almost all of the technical details in reviewing the articles. RI and AB reviewed the first draft. All authors contributed to the article and approved the submitted version.
