# Corrigendum: Eating behavior of adolescent girls in countries with a high prevalence of stunting under five: a systematic review

**DOI:** 10.3389/fpsyg.2024.1530934

**Published:** 2024-12-19

**Authors:** Arlette Suzy Setiawan, Arief Budiarto, Ratna Indriyanti

**Affiliations:** ^1^Department of Pediatric Dentistry, Faculty of Dentistry, Universitas Padjadjaran, Bandung, West Java, Indonesia; ^2^Faculty of Psychology, Universitas Jenderal Achmad Yani, Bandung, West Java, Indonesia

**Keywords:** Adolescent girls, eating behavior, nutrition, stunting, body image

In the published article, there was an error. The original text incorrectly referred to “knowledge cutoff in September 2021,” which implied the use of a generative AI model for the preparation of the manuscript. This was inaccurate and not aligned with the actual sources used in the review.

A correction has been made to **Discussion**, “*Low-diversity diet and micronutrient deficiencies*,” paragraph beginning “*Evolving Nature of the Field*.” This sentence previously stated:

“Evolving Nature of the Field: The field of nutrition and eating behaviors continually evolves. The review's findings are based on studies available up to a specific date (knowledge cutoff in September 2021). Since then, new research may have emerged that could provide additional insights or alter the conclusions.”

The corrected sentence appears below:

“Evolving Nature of the Field: The field of nutrition and eating behaviors continually evolves. The review's findings are based on studies available up to a specific date, with the most recent sources cited in this systematic review published in 2022 (Mahriani et al., 2022). Since then, new research may have emerged that could provide additional insights or alter the conclusions.”

Additionally, in the published article, there was an error in the **Acknowledgment** statement. The statement was incorrectly written as “The authors thank DRPM Universitas Padjadjaran for supporting the publication of this manuscript”. The corrected statement is:

“The authors thank DRPM Universitas Padjadjaran for supporting the publication of this manuscript. Generative AI was used in the preparation of this manuscript to enhance language clarity, coherence, and readability. Specifically, Grammarly was utilized for grammar and style checks, Smodin was employed for paraphrasing and refining certain textual elements, and ChatGPT was used to assist in drafting and restructuring sections of the manuscript.”

The authors apologize for these errors and state that they do not change the scientific conclusions of the article in any way. The original article has been updated.
